# The Endoscopic Management of Anastomotic Strictures After Esophagogastric Surgery: A Comprehensive Review of Emerging Approaches Beyond Endoscopic Dilation

**DOI:** 10.3390/jpm15030111

**Published:** 2025-03-13

**Authors:** Giuseppe Dell’Anna, Jacopo Fanizza, Francesco Vito Mandarino, Alberto Barchi, Ernesto Fasulo, Edoardo Vespa, Lorella Fanti, Francesco Azzolini, Silvia Battaglia, Francesco Puccetti, Andrea Cossu, Ugo Elmore, Antonio Facciorusso, Armando Dell’Anna, Lorenzo Fuccio, Angelo Bruni, Sara Massironi, Vito Annese, Alberto Malesci, Gianfranco Donatelli, Riccardo Rosati, Silvio Danese

**Affiliations:** 1 Gastroenterology and Gastrointestinal Endoscopy Unit, IRCCS San Raffaele Hospital, Via Olgettina 60, 20132 Milan, Italy; fanizza.jacopo@hsr.it (J.F.); mandarino.francesco@hsr.it (F.V.M.); barchi.alberto@hsr.it (A.B.); fasulo.ernesto@hsr.it (E.F.); vespa.edoardo@hsr.it (E.V.); fanti.lorella@hsr.it (L.F.); azzolini.francesco@hsr.it (F.A.); sara.massironi@libero.it (S.M.); malesci.alberto@hsr.it (A.M.); danese.silvio@hsr.it (S.D.); 2 Gastroenterology and Gastrointestinal Endoscopy Unit, IRCCS Policlinico San Donato, Piazza Edmondo Malan 2, 20097 San Donato Milanese, Italy; vito.annese@grupposandonato.it; 3 Faculty of Medicine and Surgery, Vita-Salute San Raffaele University, Via Olgettina 56, 20132 Milan, Italy; elmore.ugo@hsr.it (U.E.); rosati.riccardo@hsr.it (R.R.); 4 Gastrointestinal Surgery Unit, IRCCS San Raffaele Hospital, Via Olgettina 60, 20132 Milan, Italy; battaglia.silvia@hsr.it (S.B.); puccetti.francesco@hsr.it (F.P.); cossu.andrea@hsr.it (A.C.); 5 Faculty of Medicine and Surgery, University of Salento, Piazza Tancredi 7, 73100 Lecce, Italy; antonio.facciorusso@unisalento.it; 6 Digestive Endoscopy Unit, “Vito Fazzi” Hospital, Piazza Filippo Muratore 5, 73100 Lecce, Italy; chirurgiaendoscopica.polecce@asl.lecce.it; 7 Unit of Gastroenterology, Department of Medical and Surgical Sciences, S. Orsola-Malpighi University Hospital, University of Bologna, 40138 Bologna, Italy; lorenzo.fuccio3@unibo.it (L.F.); angelo.bruni4@unibo.it (A.B.); 8 Unité d’Endoscopie Interventionnelle, Hôpital Privé des Peupliers, Ramsay Générale de Sant, 75013 Paris, France; gianfranco.donatelli@unina.it; 9 Department of Clinical Medicine and Surgery, University of Naples “Federico II”, 80138 Naples, Italy

**Keywords:** anastomotic stricture (AS), endoscopic dilation (ED), self-expandable metal stent (SEMS), biodegradable stent (BDS), endoscopic incision technique (EIT), lumen-apposing metal stent (LAMS)

## Abstract

Anastomotic strictures are a common complication following esophagogastric surgery, with prevalence varying depending on the type of surgery and anatomical site. These strictures can lead to debilitating symptoms such as dysphagia, pain, and malabsorption, significantly impacting patients’ quality of life. Endoscopic treatment of anastomotic strictures has established a role as the first-line strategy in this setting instead of revision surgery, offering benefits in terms of lower morbidity. Various endoscopic methods are available for anastomotic stricture management, including balloon dilation, stent placement, the new lumen-apposing metal stent, and endoscopic incision techniques. However, there is currently no strong evidence and established guidelines for the optimal treatment strategy. Available data suggest that endoscopic treatments, when performed in tertiary referral centers, can provide favorable outcomes in terms of symptom relief and reduced need for rescue surgical intervention. Nonetheless, challenges remain regarding the management of recurrent strictures and procedural complications, underscoring the need for a personalized, multidisciplinary approach to optimize clinical outcomes. This review aims to provide an updated overview of endoscopic techniques and available evidence with a focus on the most recent technologies, supporting clinicians in effectively managing anastomotic strictures in complex clinical settings.

## 1. Introduction

**Figure 1:**
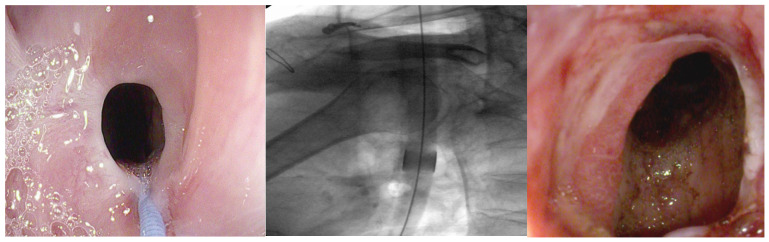
Stenosis of the esophagogastric anastomosis treated with a Savary–Gilliard dilator. The copyright of this figure belongs to the authors.

**Figure 2:**
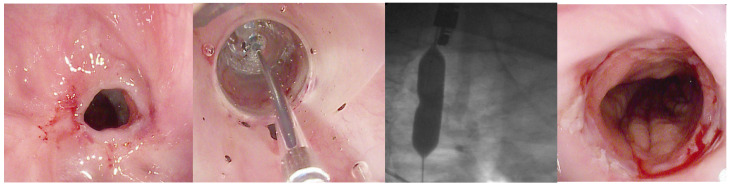
Stenosis of the esophagogastric anastomosis treated with a through-the-scope balloon dilator. The copyright of this figure belongs to the authors.

Anastomotic strictures (ASs) represent a relatively common and challenging complication following gastroesophageal surgery, such as esophagectomy, gastrectomy, and other types of oncologic or functional surgical procedures [1]. These strictures are caused by a scarring process that leads to the narrowing of the anastomotic lumen, hindering the passage of food content. Their incidence varies depending on multiple factors, including the surgical technique employed, the surgeon’s experience, the presence of infections or dehiscences in the postoperative phase [2,3], the use of adjuvant treatments such as radiotherapy [4], and patient comorbidities (diabetes or cardiovascular diseases) [4,5]. Esophagogastric ASs occur in 0.5% to 42% of patients following esophageal resection [4], while the reported incidence of gastrojejunal (GJ) AS following laparoscopic Roux-en-Y gastric bypass (L-RYGB) varies widely in the literature, ranging from 0.8% to 27% [6]. Anastomotic stenosis typically manifests with progressive dysphagia, weight loss, and occasionally regurgitation or epigastric pain. Diagnosis relies on a combination of patient history and symptom assessment, endoscopic evaluation, and radiological imaging, such as contrast-enhanced radiography. Endoscopy plays a crucial role in both diagnosis and treatment, with endoscopic dilation (ED) being the primary intervention [7,8]. ED represents the traditional first-line treatment for benign esophageal strictures [9,10]. Various types of ED can be performed: mechanical with bougie and pneumatic with balloon-type dilators. Mechanical dilators are further subdivided based on the need for guidewire placement and/or fluoroscopic guidance to complete the procedure. Non-guidewire-dependent bougies, such as Maloney dilators, are filled with mercury or tungsten and feature a tapered tip. They are available in a variety of sizes to suit different clinical needs. [11]. The most widely utilized guidewire-assisted mechanical bougie is the polyvinyl Savary–Gilliard dilator (Cook Medical Device Company, Billerica, MA, USA). Through-the-scope balloon dilators (BD) are available in both guidewire-assisted and non-guidewire-assisted variants. Among the available options, the Savary–Gilliard bougie (Figure 1) and BD (Figure 2) remain the most commonly employed in clinical practice [11].

**Figure 3:**
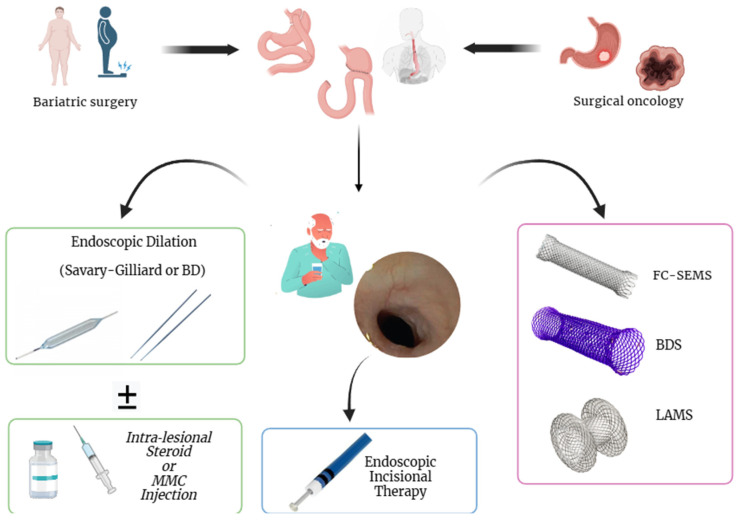
Endoscopic treatments available for the management of GI anastomotic stenosis resulting, for example, from bariatric or oncological surgery. BD, balloon dilator; MMC, mitomycin C; FC, fully covered; SEMS, self-expanding metal stent; BDS, biodegradable stent; LAMS, lumen-apposing self-expanding metal stent. The copyright of this figure belongs to the authors.

While there are mechanistic differences between balloon and bougie dilation, no clear advantage of one over the other has been conclusively demonstrated. A 2018 meta-analysis by I. R. Josino et al. [12]. compared ED using bougies versus BD for benign esophageal strictures, including AS [12]. In terms of postprocedure symptomatic relief, the study found no significant difference between the groups, with I^2^ = 0% (95% CI [−0.08, 0.08]) [12]. The 12-month recurrence rate showed a risk difference of 0.03 (95% CI [−0.05, 0.10]) and a heterogeneity of I^2^ = 59%. Even after reanalyzing the pooled data, heterogeneity decreased to I^2^ = 20%, yet no significant difference between the methods was detected. No differences in perforation rate and hemorrhage rate were observed [12]. A retrospective study by A. H. Mendelson et al. [13]. on only 74 patients with esophageal AS (after esophagectomy and laryngectomy) evaluated the outcomes of ED over five years [13]. Clinical success (CS), defined as maintaining a luminal diameter ≥14 mm for at least four weeks, was observed in 93% of patients. Despite this, recurrence occurred in 43% of cases, with a median time to recurrence of 152 days (IQR: 49–312) and a median of two additional dilations required for reestablishing patency [13]. Instead, in a retrospective study of 751 patients with benign esophageal stenosis (BES), Vermeulen et al. [14]. analyzed 416 cases of anastomotic stenosis, with patients undergoing an average of 4.9 ± 2.3 EDs to assess their risk factors and clinical outcomes [14]. Among 390 patients included in the risk factor analysis for refractory stenosis [median follow-up 39 months (IQR, 17–80), and median time to the first ED after esophagectomy 91 days (IQR, 56–182)], risk factors associated with requiring more ED sessions included: first ED within 90 days postesophagectomy (mean 6.6 vs. 3.7 sessions; HR 1.8; *p* < 0.001), preoperative cardiovascular disease (mean 5.3 vs. 4.9 sessions; HR 1.5; *p* = 0.002) and previous anastomotic leakage (mean 6.1 vs. 4.7 sessions; HR 1.3; *p* = 0.037) [14]. No significant associations were found between the mean number of ED sessions and initial stricture diameter (<10 mm vs. ≥10 mm; *p* = 0.297) or anastomosis location (cervical vs. intrathoracic; *p* = 0.243) [14]. These findings highlight the high recurrence and refractoriness of anastomotic strictures despite successful initial dilation, emphasizing the need for optimized treatment strategies and adjunctive therapies to improve long-term outcomes also based on patient risk factors. [13]. In general, approximately 10% of patients undergoing dilation for gastrointestinal (GI) strictures do not achieve complete resolution and require repeated dilations [15]. While most patients are effectively treated with up to five dilation sessions, some develop refractory or recurrent strictures, as defined by the Kochman criteria [13,14]. Refractory or recurrent strictures refer to anatomical restrictions caused by cicatricial luminal narrowing or fibrosis, resulting in clinical symptoms of dysphagia without endoscopic evidence of inflammation [14]. Refractory strictures occur when it is not possible to achieve a luminal diameter of at least 14 mm after five dilation sessions at two-week intervals. Recurrent strictures, on the other hand, arise when it is not possible to maintain a satisfactory luminal diameter for four weeks after achieving the target diameter of 14 mm [14]. Advances in endoscopic therapy for benign luminal stenosis, including those caused by surgical procedures, chemical or radiation injury, and other etiologies, have significantly broadened the available treatment options beyond traditional dilation [7]. However, clear guidelines for determining the optimal therapeutic approach, whether endoscopy surgery or endoscopic dilation (ED) versus other endoscopic techniques remain lacking. Therefore, the goal of this review is to offer an updated analysis of the various therapeutic options available beyond ED for managing anastomotic stenosis, emphasizing the most recent findings in the literature. Figure 1 illustrates all the available endoscopic treatment options (Figure 3).

## 2. Materials and Methods

In our review, we conducted a comprehensive search across PubMed, Scopus, Web of Science, and Medline, including only English-language articles published up to the end of January 2025. Our search strategy utilized detailed search strings incorporating the following terms: “anastomotic stricture”, “esophagectomy”, “gastrectomy”, “esophagogastric surgery”, “post-surgical anastomotic stenoses”, “endoscopic dilation”, “intra-lesional steroid injection”, “MMC”, “self-expandable metal stents”, “biodegradable stents”, “endoscopic incision techniques”, and “Lumen-Apposing Metal Stent”. Additionally, we manually screened the references of the included studies and relevant reviews to identify any further eligible publications.

## 3. Endoscopic Technique

### 3.1. Intra-Lesional Steroid Injection (ILSI)

ILSI and mitomycin C (MMC) injections or topical applications are currently considered the only available adjunctive treatments for ED, with the rationale to inhibit the inflammatory response following dilation [7]. This result is achieved by reducing collagen production, thereby minimizing fibrosis and chronic scarring [7]. The effectiveness of combination therapy involving ILSI and ED has been demonstrated in improving clinical outcomes in other types of strictures, such as peptic [16,17] or post-ESD ones [18,19]. However, the underlying mechanisms of anastomotic and, for example, peptic strictures differ significantly. Peptic strictures result from inflammation and ulceration caused by gastric acid reflux, whereas AS are primarily attributed to ischemia of the proximal gastric tube [20]. Despite this difference, both types involve chronic inflammation of esophageal mucosa. Based on this similarity, it has been hypothesized that combining corticosteroid injections with ED for AS could reduce the frequency of repeated dilations and extend the dysphagia-free interval. Furthermore, there is a lack of standardization and consistency regarding the appropriate dosage and treatment protocols in existing studies [21]. In a multicenter, double-blind trial, Hirdes MMC et al. [22]. randomized 60 patients with naive cervical AS following esophagectomy with gastric tube reconstruction and dysphagia [22]. The patients were assigned to receive four-quadrant injections of either 0.5 mL triamcinolone (40 mg/mL; steroid group, *n* = 29) or saline (control group, *n* = 31) directly into the stricture, followed by Savary ED to 16 mm. After six months of follow-up, 45% of steroid group patients remained free from dysphagia, compared to 36% of control group patients who received saline injections (RR: 1.26; 95% CI: 0.68–2.36; *p* = 0.46). The median time to repeat dilation was 108 days (range, 15–180) in the steroid group, compared to 42 days in the control group (log-rank test, *p* = 0.29) [22]. The median number of dilations performed within six months was two in the steroid group and three in the saline group (RR: 0.76; 95% CI: 0.42–1.37; *p* = 0.36). The major AEs observed during follow-up were associated with ED. In the corticosteroid group, four patients developed Candida esophagitis proximal to the anastomosis, compared to none in the saline group (*p* = 0.03) [22]. Contrasting results come from the small retrospective series by J. C. Pereira-Lima et al. [23]., including 19 patients with naïve AS (impassable with a 0.98 cm endoscope) after esophagectomy with gastric conduit (hand-stitched cervical esophagogastrostomy) [23]. Highlighting the lack of standardization in corticosteroid injection treatments within the literature, this study randomized patients to receive either a total of 40 mg of triamcinolone acetonide, injected around and at the edges of the lacerations caused by bougie dilation after each therapeutic endoscopy session (*n* = 10), or saline injections following bougie dilation (*n* = 9) [23]. At the 6-month follow-up visit, 62% of steroid group patients were free of dysphagia, compared to 0% in the control group (*p* = 0.009), without any AEs observed in the steroid group [23]. A subsequent randomized controlled trial (RCT) included patients with dysphagia caused by AS following subtotal transthoracic esophagectomy for esophageal cancer with gastric conduit reconstruction, naïve to prior endoscopic treatment [24]. Sixty-five consecutive patients underwent ED using a standard through-the-scope balloon dilator and were then randomized to receive either a total of 50 mg of triamcinolone acetonide (*n* = 33) or an equivalent volume of normal saline solution (*n* = 32) [24]. The median number of ED sessions required to solve the stricture was 2.0 (interquartile range [IQR], 1.0–2.5) in the steroid group compared to 4.0 (IQR, 2.0–6.8) in the placebo group (*p* < 0.001). At the 6-month follow-up, 39% of steroid group patients were free of symptom recurrence, compared to 16% in the placebo group (*p* < 0.01) [24]. The median time to stricture resolution was 22 days (IQR, 0–72 days) for the steroid group and 92 days (IQR, 34–176 days) for the placebo group (*p* < 0.001). No AEs occurred [24].

### 3.2. MMC Topical Application or Injection

MMC is an anthracycline chemotherapy drug derived from *Streptomyces*, known for its ability to inhibit RNA and protein synthesis [25]. MMC is recognized as both an antibiotic and an antineoplastic agent that suppresses fibroblast proliferation, thereby influencing the healing process [26]. It has been effectively used as an antifibrotic agent in both ophthalmologic and pulmonary diseases [27]. Its application in treating GI tract stenosis, particularly anastomotic stenosis following esophagogastric surgery, remains constrained due to the limited number of studies available. Moreover, existing evidence varies in the populations treated and addresses GI stenosis of a different nature. The largest available study on the role of topical MMC application in treating benign esophageal strictures was published in 2015 by Zhang et al. [28]. The authors compared the combined use of MMC and ED with ED and local dexamethasone injection and ED alone [28]. In this study, the patients were divided into three groups: the MMC intramuscular injections group, the dexamethasone intramuscular injections group, and the saline intramuscular injections group [28]. AS were observed in 64%, 72%, and 66.7% of patients in the MMC, dexamethasone, and dilation-only groups, respectively, while the remaining portion consisted of post-ESD strictures. The median of the prior dilation sessions in each group was 3 (range 1–5 in MMC, and only the ED group and 1–4 in the dexamethasone group) [28]. All patients achieved technical and CS after ED, and no major AEs, such as significant bleeding, leakage, or perforation, were reported in any group [28]. After discharge, all patients received clinical follow-up for more than 6 months. During the follow-up, the MMC group experienced the longest dysphagia-free period (4.88 ± 1.66 months), followed by the dexamethasone group (4.02 ± 1.77 months), with the conventional dilation group showing the shortest duration (2.41 ± 1.26 months) [28]. A study by M.J. Bartel et al. [27]. evaluated the use of MMC in a small cohort of nine patients with different types of esophageal strictures, including anastomotic, radiation-induced, caustic, and combined forms [27]. Before inclusion, they underwent an average of 10.7 ED over a median of 8 months. Seven out of nine (77.7%) had prior steroid injections, 4/9 (44.4%) had stent placements, and 3/9 (33.3%) received EIT. At baseline, the median dysphagia score was 3 (range 1–4) [27]. The results showed that MMC, when used alongside ED or advanced endoscopic techniques, may help decrease the frequency of dilation procedures required for recurrent benign complex strictures [27]. After MMC application with ED, the mean periodic dilation index (PDI) significantly decreased from 1.53 to 0.39 (*p* = 0.01), while the dysphagia score showed a nonsignificant reduction from 3.2 to 2.6 at the last follow-up [27].

The role of MMC in managing GI strictures, particularly postsurgical ones, remains poorly studied. Although MMC has shown the potential to complement ED by reducing the frequency of treatments, significant uncertainties persist. Prospective studies are lacking, and no consensus exists on critical factors such as dosage, injection technique (submucosal versus intramuscular), or administration protocols. While MMC is generally considered safe, its classification as a chemotherapy agent necessitates special certification at some institutions [7]. Given the limited data on its safety and long-term effects, careful patient monitoring is essential [29].

### 3.3. Endoscopic Incision Technique (EIT)

The EIT involves excising the fibrotic tissue forming the stricture, typically using electrocautery, along the longitudinal axis. The EIT for treating benign AS was first reported in two small-size studies. In 2002, the EIT was proposed by G. Brandimarte and A. Tursi as the primary treatment for six consecutive patients with esophagus-jejunal AS [30]. Six radial incisions were performed using the sphincterotome. Technical success (TS) and CS, defined as the resolution of the stenoses without any procedure-related complications, were achieved in all cases. The patients were monitored for a period ranging from 8 to 33 months (median follow-up of 24 months), and no recurrence of stenosis was observed [30]. In 2009, T.H. Lee et al. [31] evaluated, in a prospective study, the EIT as a first-line treatment in 24 patients with AS after esophagogastric oncological surgery. Both the Iso-Tome (used in 16 patients) and the insulated-tip (IT) knife (used in eight patients) were utilized, and stricture dilation was successfully achieved in all patients within a single treatment session. On average, nine radial incisions were performed per patient (range, 8–12), all completed successfully [31]. After 24 months of follow-up, 21/24 (87.5%) of patients were free from dysphagia and endoscopic evidence of recurrence. Furthermore, no procedural-related AEs were registered during follow-up [31]. Just 2/24 (8.3%) patients achieved resolution with a single additional dilation session among those with a recurrence; 1/24 (4.1%) patients were refractory and underwent five EITs [31]. In 2006, M. L. Hordijk et al. [32] applied the EIT to treat AS unresponsive to ED, with no reported complications [32]. Among 12 to 20 patients with strictures shorter than 1 cm, dysphagia was resolved after a single treatment. However, in all eight patients with long-segment strictures measuring 1.5 to 5 cm, dysphagia recurred, requiring an average of three treatment sessions to achieve resolution [32]. A recent meta-analysis by Z. Jimoh et al. [33], published in 2024, included five studies comparing the EIT with ED (using Savary–Gilliard bougies or BD) [33]. Two studies involved patients with both esophagogastric and esophagojejunal AS. Another two studies focused exclusively on patients with esophagogastric AS, while one study included only those with esophagojejunal AS [33]. The EIT technique was consistent across the included studies, involving radial incisions with cautery using either an IT knife or a hook knife. In two studies, these radial incisions were connected circumferentially around the fibrotic tissue forming the stricture, while three studies did not perform circumferential connections. For the dilation procedure, four studies employed EBD, whereas one study used Savary–Gilliard bougie dilation [33]. A combined meta-analysis of naïve and recurrent strictures showed significantly reduced odds of stricture recurrence in patients treated with EIT compared to ED (OR 0.35, 95% CI 0.13–0.92, *p* = 0.03; I^2^ = 71%). A subgroup analysis of four studies focusing on naïve strictures also revealed significantly lower odds of stricture recurrence with EIT compared to ED (OR 0.32, 95% CI 0.17–0.59, *p* = 0.0003; I^2^ = 0%) [33]. However, a subgroup analysis of three studies examining recurrent strictures found no significant difference in stricture recurrence odds between the two groups (OR 0.63, 95% CI 0.12–3.28, *p* = 0.58; I^2^ = 81%). Additionally, this meta-analysis revealed significantly lower odds of stricture recurrence with EIT compared to ED for both naïve and recurrent strictures combined (OR 0.35, 95% CI 0.13–0.92, *p* = 0.03; I^2^ = 71%). Subgroup analysis of naïve strictures showed a similar significant benefit with EIT (OR 0.32, 95% CI 0.17–0.59, *p* = 0.0003; I^2^ = 0%) [33]. However, for recurrent strictures, no significant difference was observed between EIT and ED (OR 0.63, 95% CI 0.12–3.28, *p* = 0.58; I^2^ = 81%). Three studies found no significant difference in complication rates between the two groups [34,35,36]. One study reported a significantly higher perforation rate in patients undergoing ED compared to incisional therapy (38,1% vs. 28% respectively) [37]. In comparison, another study observed a higher perforation rate in patients treated with EIT compared to ED (6.3% vs. 0% respectively) [38].

### 3.4. Endoscopic Stents

**Figure 4:**
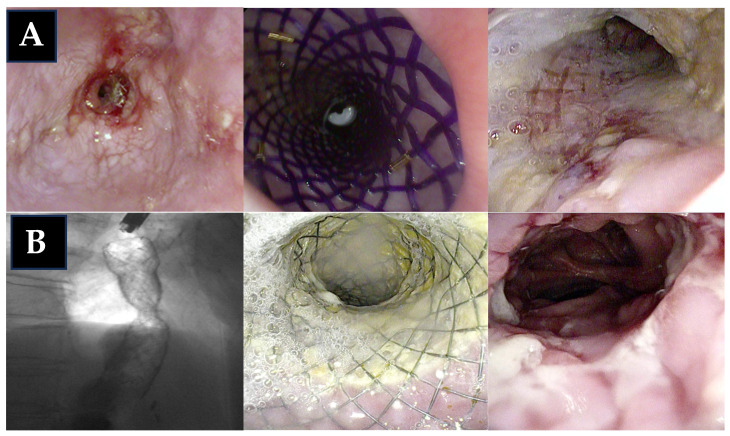
Refractory postsurgical stenosis treated with the placement of biodegradable stents (**A**) and with a fully covered self-expanding metal stent (**B**). The copyright of this figure belongs to the authors.

Endoscopic stents play a pivotal role not only in the management of anastomotic fistulas following esophagogastric surgery but also in the treatment of refractory AS [39,40]. Stent placement is a commonly used strategy for managing refractory benign GI stenosis [41], particularly in cases that remain unresponsive after at least five sessions of endoscopic balloon dilation (EBD) over two weeks and fail to resolve the stenosis [42]. Various types of stents are employed in this setting, including self-expanding metal stents (SEMSs) and biodegradable stents (BDSs) (Figure 4). Currently, the two most used SEMS models are fully covered SEMSs (FC-SEMSs) and partially covered SEMSs (PC-SEMSs). FC-SEMSs have a plastic or silicone layer that entirely covers the metal mesh, whereas PC-SEMSs have uncovered proximal and distal ends [39]. This design helps prevent migration but may encourage mucosal ingrowth. In the past, self-expandable plastic stents (SEPSs) were also utilized. However, complications such as stent migration with SEPSs and PC-SEMSs have led to a decline in their use for refractory benign GI strictures [42,43]. In the last decades, the lumen-apposing metal stent (LAMS), a dumbbell-shaped, short SEMS, originally designed for therapeutic endoscopic ultrasound (EUS) procedures (such as EUS-biliary drainage, EUS-gastroenterostomy, EUS-guided pancreatic fluid collection drainage), emerged as a new valid device in AS management [44,45,46].

Available evidence on the role of endoscopic stents in this setting exhibited significant heterogeneity, particularly concerning the characteristics of patients included (e.g., varying types of stenoses) and the treatment modalities employed (e.g., first-line treatment versus refractory stenoses). In this section, we have highlighted the most relevant studies focusing on the use of endoscopic stents for postsurgical stenoses.

#### 3.4.1. Fully Covered-Self Expandable Metal Stents (FC-SEMSs)

FC-SEMSs were designed with a synthetic outer layer, typically composed of silicone or polyurethane derivatives, to mitigate the high re-intervention rates associated with uncovered SEMSs, which are prone to obstruction caused by tumor ingrowth or inflammatory tissue proliferation [47]. The coating serves to prevent the stent from embedding into the wall and inhibits tissue growth within the lumen [48]. An additional advantage of FC-SEMSs is their ease of removal, which can be performed using endoscopic and/or fluoroscopic guidance. On the other hand, the main limitation of the FC-SEMS is the higher risk of migration compared to uncovered ones [49]. To prevent migration, various fixation or anchorage techniques can be used, including external snare fixation, endoscopic suturing [50], and endoscopic clips, either through-the-scope (TTS) or over-the-scope (OTSC) [51], resulting in reduced stent migration rates [52].

The role of the FC-SEMS in managing GI-AS was evaluated by J. Liu et al. in 24 patients with AS after esophagectomy for esophageal cancer [53]. All patients met Kochman’s criteria for refractory stenosis, having undergone at least five sessions of ED without symptom improvement. A total of 29 FC-SEMSs were successfully placed and removed within 4–8 weeks. Stent migration occurred in one asymptomatic patient, and no FC-SEMS-related AEs were registered during the study period [53]. CS, defined as an improvement in the dysphagia scoring system, was reached in all patients. Recurrence occurred in 7/24 (29%) patients within three months, five of whom underwent restenting, but only one achieved lasting symptom relief. After 12 months, 18/24 (75%) patients remained free of dysphagia, while six experienced persistent symptoms [53]. FC-SEMSs have previously been evaluated for the management of anastomotic stenosis as part of broader studies involving diverse patient populations and clinical conditions. Indeed, J.C. Bakken et al. [54] and M.A. Eloubeidi et al. [55] had previously evaluated the application of FC-SEMSs in the management of benign esophageal pathology. In the study by Bakken et al. [54], 10 patients with surgical anastomotic strictures after esophagectomy were treated with FC-SEMS placement [54]. Five out of ten (50%) patients showed CS, defined as either the resolution or improvement of dysphagia symptoms, as documented in clinical follow-up notes, radiologic or endoscopic studies confirming stricture resolution, or both at the time of stent removal. However, 3/10 (30%) of these patients experienced recurrence within 60 days, and long-term follow-up data was unavailable for the remaining two [54]. The migration rate for anastomotic stenosis was 60%; however, data on other complications specific to this patient group are unavailable [54]. Eloubeidi et al. [55] conducted a retrospective analysis of patients referred for the management of benign esophageal disease, in which 7/35 patients (20%) with an anastomotic stricture underwent placement of an FC-SEMS [55]. A total of 11/35 patients (31%) achieved successful treatment, defined as the resolution of presenting symptoms with a lasting response after stent removal. However, no specific details about these patients were provided. Stent migration occurred in 12 patients [55]. Migration was more common, though not statistically significant, in stents placed for strictures (7/19, 37%) and fistulae/leaks (4/12, 33%) compared to those placed for perforations (1/4, 25%), with *p*-values of 0.614 and 0.339, respectively [55]. In a multicenter retrospective study conducted by T. Suzuki et al. [56], the efficacy and safety of FC-SEMS were assessed in cases of benign esophageal stenosis [56]. Among the 70 patients included, 13 (18.5%) had anastomotic stenosis, although details regarding the type of surgery, surgical indication, and type of anastomosis were not available. Within this subgroup, 23 FC-SEMS placements were performed [56]. Treatment success, defined as the resolution of dysphagia without the need for restenting following final stent removal, was achieved in only three patients (3/13, 23.1%), while 12 patients (92.3%) required restenting. The mean number of treatment sessions with stenting was 2 (range 1 to 7). Stent migration was observed in 8/23 procedures (34.8%) and in 5/13 patients (38.5%) [56].

#### 3.4.2. Biodegradable Stents (BDSs)

BDSs are stents typically composed of bioabsorbable materials such as polydioxanone (PDO), a material commonly used in surgical sutures, poly-L-lactic acid (PLLA), or magnesium [57]. These stents gradually degrade over time through biological mechanisms such as hydrolysis, a process that is accelerated in acidic environments. Degradation generally begins within 4 to 5 weeks, with complete dissolution occurring over 2 to 3 months [58]. A key advantage of BD stents compared to SEMSs or SEPSs is that they do not require removal, even in cases of migration. Instead, they remain in place until fully dissolved by gastric acid, which accelerates hydrolysis, thereby eliminating the need for additional procedures and reducing potential morbidity. Currently available BD stents include the ELLA-BD stent (ELLA-CS, Hradec Kralove, Czech Republic), constructed from polydioxanone, a material used in surgical sutures, and the PLLA-BD stent (Marui Textile Machinery, Osaka, Japan), composed of knitted PLLA monofilaments [57]. The development of the BDS is based on the rationale that they can exert a constant radial force for a defined period (4–5 weeks), allowing sufficient time to address the underlying esophageal condition [58]. Repici et al. [59] assessed the safety and efficacy of BDS for managing refractory benign esophageal strictures in a cohort of 21 patients, including five cases of AS [59]. All participants had previously undergone at least one ED session, and the mean length of the strictures was approximately 3 ± 1 cm [59]. CS, defined as the resolution of dysphagia without recurrence during the follow-up, was observed in two of the five patients with AS, while the remaining cases required at least one additional ED session. No BDS-related AEs were documented [59]. Jeanin E. van Hooft et al. [60] investigated the application of BDS for the management of naïve AS in 10 patients, nine of whom had previously undergone esophagectomy for esophageal carcinoma, while one had undergone the procedure for Boerhaave’s syndrome [60]. In 6/10 cases (60%), BDS provided effective resolution as a one-step treatment, with no reinterventions required during the 6-month follow-up period [60]. However, one patient experienced food impaction 74 days after stent placement, necessitating an endoscopy without any signs of pathological narrowing or stent-related complications in the esophagus. The remaining 15 weeks of follow-up were uneventful. Additionally, two patients developed obstruction due to hyperplasia, and one patient experienced a recurrence of the AS [60]. Notably, no other AEs, such as perforation, bleeding, stent migration, or GI obstruction related to stent migration, were observed [60]. More robust data come from the RCT by D. Walter et al. [61], published in 2018, evaluating the efficacy and safety of BDS in managing benign GI strictures, particularly postsurgical ones. The study involved 66 patients: 32 randomized in the BDS group and 34 in the ED group [61]. Most patients in both groups had AS, 26/34 (76.5%) in the dilation group and 23/32 (71.9%) in the stent group, while 11 patients in the BDS group underwent previous ED to 16 mm within the prior year. Primary outcomes included the frequency of repeat ED for recurrent strictures within 3 and 6 months after the intervention [61]. At 3 months, patients in the BDS group required significantly fewer repeat dilations compared to the dilation group (median 0 vs. 1; *p* < 0.001). By 6 months, no difference was noted between the groups (median 1 vs. 1; *p* = 0.31). The probability of not needing ED was significantly higher in the BDS group compared to the ED group across various follow-up periods, with 87.5% at 3 months, 48.4% at 6 months, and 40.8% at 12 months, compared to 49.5%, 34.1%, and 27.9%, respectively, in the dilation group (log-rank *p* = 0.05) [61]. Additionally, the median time to the first re-ED was significantly longer in the BDS group compared to the ED group (106 days vs. 41.5 days; *p* = 0.003). No differences according to AE rate were recorded (*p* = 0.42), with the most frequently reported AE being recurrent significant dysphagia requiring intervention. In the BDS group, complications included stent occlusion (5/32, 15.6%), tracheoesophageal fistula (2/32, 68.7%), and stent migration (1/32, 3.1%) [61].

#### 3.4.3. Lumen-Apposing Metal Stent (LAMS)

LAMSs are biflanged dumbbell-shaped FC-SEMSs initially developed to facilitate EUS-guided transluminal drainage, commonly used in the management of pancreatic pseudocyst and walled-off pancreatic necrosis [46,62,63,64]. Over time, LAMSs were largely used for additional indications, including biliary drainage in distal malignant biliary obstruction and in patients with surgically altered anatomy, gallbladder drainage in unfit-for-surgery patients, and the management of malignant gastric outlet obstruction [45,65,66,67,68]. In clinical practice, the application of the LAMS has expanded beyond the approved indications to include uses such as the treatment of benign GI luminal strictures [43]. LAMSs offer several advantages, including the availability of variable diameters and lengths, a saddle-shaped design with wide flanges that enhance anchorage and minimize migration risk and a straightforward, stepwise deployment process that ensures high TS rates (Table 1). Another important factor is the radial force exerted by the LAMS as it gradually expands during full deployment. Limited evidence is available now on this topic but LAMSs showed promising results [69]. The role of the LAMS in treating benign GI stenosis was assessed in a meta-analysis published in 2023 by S. Giri et al. [69], including 18 studies, of which only two were prospective [69]. The proportion of patients with AS varied between 40% and 100%. Most patients suffered from AS refractory to the other endoscopic treatments, including ED with or without ILSI, placement of a FC-SEMS, or stricturotomy. The median stent dwell time varies from 60 to 119 days [69]. The authors also performed a subgroup analysis, including only patients with AS. In this setting, the pooled TS rate of LAMS placement was 99.9% (95% CI 99.1–100.0), demonstrating an almost perfect ability to position the stent across the stricture. The short-term CS rate, reflecting symptom resolution or improvement with the stent in place, was 94.6% (95% CI 90.6–98.7). Long-term CS, which evaluates symptom resolution after stent removal without additional interventions, was slightly lower at 76.9% (95% CI 61.1–92.8). The pooled event rates revealed no significant differences compared to the overall data for all benign GI stenoses, with a TS rate of 99.9% (95% CI: 99.1–100), a short-term CS rate of 93.9% (95% CI: 90.7–100), and a long-term CS rate of 72.8% (95% CI: 55.7–90.0) [69]. Across studies, the pooled incidence of AEs in benign GI strictures was 13.5% (95% CI: 8.6–18.5), with common complications including pain (5.7%), bleeding (2.3%), infection (0.9%), and stent occlusion (1.8%). Migration rates for benign GI strictures were reported at 10.6% (95% CI: 6.0–15.2). For AS, the AE rate was slightly higher at 14.4% (95% CI: 7.1–21.6), while the migration rate increased to 13.3% (95% CI: 6.5–20.0) [69]. On the other hand, a prospective study by Skidmore et al. [70] assessed the use of LAMSs as a first-line approach for managing AS. The study evaluated the use of LAMSs in 14 patients with gastrojejunostomy strictures following Roux-en-Y gastric bypass (RYGB) [70]. These patients, representing 3.3% of the 421 individuals who underwent RYGB during the study period, were treated with LAMSs for symptomatic AS. Of these, 12 patients achieved complete resolution of their strictures, resulting in a TS rate of 100% and a CS rate of 85.7%. The stents were left in place for an average duration of 44 days (range: 10–161 days) [70]. A total of 26 stents were placed, with 19% experiencing distal migration; however, no additional interventions were needed for their removal. Two patients required surgical revision due to refractory strictures associated with marginal ulcers. No immediate AEs were observed, further supporting the safety and efficacy of the LAMS in managing AS post-RYGB [70].

Notably, no studies have directly compared LAMSs with the current therapeutic standard of ED. Conversely, a meta-analysis evaluated and compared the efficacy and safety of various endoscopic stents, including the FC-SEMS, BDS, and LAMS, in the management of benign GI stenoses [43]. The TS rate was similar between LAMSs and FC-SEMSs. However, the BDS exhibited the lowest TS rate (91.9%) compared to the LAMS (97.6%, *p* = 0.05) and FC-SEMS (96.5%, *p* = 0.06). This difference may be attributed to variations in stent delivery technology [43]. The clinical success (CS) rate was significantly higher for the LAMS, with a pooled rate of 78.8% (95% CI: 65.8–87.8; 95% PI: 28.1–97.3; I^2^ = 69.6), compared to 48.4% for FC-SEMS (95% CI: 37.1–59.8; 95% PI: 14.5–83.4; I^2^ = 70.4) and 34.9% for BDS (95% CI: 23.6–48.1; 95% PI: 11.2–69.5; I^2^ = 55.5) [43]. The differences between the LAMS and FC-SEMS (*p* = 0.001), as well as between LAMSs and BDSs (*p* = 0.001), were statistically significant, whereas no significant difference was observed between FC-SEMS and BDS (*p* = 0.12) [43]. A key finding to highlight is the stent migration rate. LAMSs exhibited the lowest migration rate, with a pooled value of 13.7% (95% CI: 9.2–20.0; 95% PI: 8.3–21.9; I^2^ = 0), compared to 31.5% for FC-SEMSs (95% CI: 26.1–37.5; 95% PI: 18.5–48.3; I^2^ = 27.8) and 11.5% for BDS (95% CI: 7.4–17.3; 95% PI: 7.0–18.4; I^2^ = 0) [43]. The differences between LAMSs and FC-SEMSs (*p* = 0.001) and between FC-SEMSs and BDSs (*p* = 0.001) were statistically significant, while no significant difference was observed between LAMS and BDS. These findings underscore the high clinical efficacy of LAMSs, combined with a relatively low migration rate compared to FC-SEMSs, making them a preferable choice in terms of overall performance [43]. No statistically significant differences in bleeding rates were observed among the three groups: 4.9% for LAMSs (95% CI: 2.5–9.3, I^2^ = 0), 2.6% for FC-SEMSs (95% CI: 1.3–5.1, I^2^ = 0), and 5.5% (95% CI: 3.0–10.0, I^2^ = 0) [43]. A total of four perforation cases were reported across all patients, with one occurring in the LAMS group and three in the BDS group [43].

## 4. Discussion

**Figure 5:**
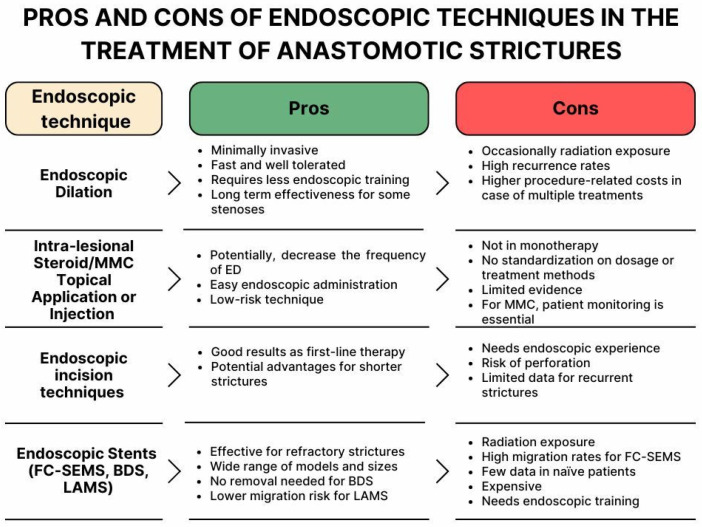
Pros and cons of all endoscopic techniques used in the treatment of GI anastomotic strictures. MMC, mitomycin C; FC, fully covered; SEMS, self-expanding metal stent; BDS, biodegradable stents; LAMS, lumen-apposing self-expanding metal stent. The copyright of this figure belongs to the authors.

AS, together with anastomotic leak and delayed gastric conduit emptying, represent the more common complications after esophagogastric surgery, for which endoscopy plays a key role in the treatment that remains challenging [71,72]. Among the available therapeutic options, ED has long been regarded as the first-line treatment due to its standardized protocols and minimally invasive nature. However, its high recurrence rates often require multiple sessions, leading to increased cumulative costs and potential procedural risks. The most commonly reported AEs following ED for benign conditions include perforation, hemorrhage, and bacteremia, with the risk of perforation estimated to range between 0.1% and 0.6% per procedure [73]. The use of endoscopic techniques, such as ILSI and MMC injection, in combination with ED, represents an initial effort to reduce the number of required endoscopic sessions, aiming to minimize patient risks and reduce traditional management costs. However, available data on the use of ILSI and MMC injections for treating ASs remain limited, making it challenging to develop standardized protocols for administration techniques and appropriate dosing. In fact, there is a lack of prospective studies, and no consensus has been established on several key factors. Steroid dosages, for instance, vary widely across studies, ranging from 40 mg to 50 mg and even up to 80 mg. Furthermore, the injection technique is not standardized, with options including submucosal or intramuscular injections. The administration protocol also remains unclear, with variations on whether the treatment should be given before or immediately after complementary endoscopic procedures. Additionally, while most studies focus on ED, there is no available data on combining this treatment with EIT or stent placement. Furthermore, their role in managing postsurgical GI strictures remains underexplored, with existing studies often being heterogeneous and involving diverse patient populations. It is also important to recognize that different types of strictures—such as anastomotic, post-ESD, and peptic strictures—are driven by distinct pathophysiological mechanisms. Consequently, ILSI may be more effective in strictures with an inflammatory basis, such as peptic strictures, whereas its use in radiation-induced strictures is discouraged by some experts due to reported cases of fistula formation [7]. Additionally, considering its safety profile and cost-effectiveness, recent guidelines recommend limiting ILSI to no more than three sessions before exploring alternative treatment strategies [7]. Findings in the literature suggest that EIT is a valid and effective therapeutic option for managing AS, particularly in naïve cases. The significant reduction in recurrence rates compared to ED, as highlighted by the meta-analysis, emphasizes the potential advantages of the incisional approach, especially for shorter strictures [33]. However, in recurrent or longer strictures, the efficacy of EIT appears to be limited, often necessitating multiple sessions to achieve resolution. An analysis of complication rates does not indicate significant differences between EIT and ED, although some studies report higher perforation rates in one group or the other, depending on the specific study [33]. This aspect warrants further investigation to optimize the selection of therapeutic techniques based on patient profiles and stricture characteristics. While EIT has been proposed as a first-line alternative to ED, endoscopic stents—such as FC-SEMS, BDS, and LAMS—represent valuable options for managing anastomotic stenosis, particularly in cases refractory to repeated ED. However, the heterogeneity in patient populations and treatment protocols across studies complicates the assessment of their efficacy as a standardized approach. FC-SEMSs are a widely used option due to their ease of placement and removal, along with their ability to prevent tissue ingrowth through their synthetic coating. However, their use in anastomotic stenosis is limited by high migration rates, reported at 31.5% in meta-analyses, and moderate CS rates compared to newer devices such as LAMSs [43]. BDSs offer the advantage of eliminating the need for endoscopic removal, as they naturally degrade over time. However, their CS rate in treating anastomotic stenosis remains low, with reported complications including tissue hyperplasia and partial stent occlusion [60]. LAMSs have emerged as a promising alternative, particularly used in refractory cases. Their unique saddle-shaped design together with the short stent body, reduces the risk of migration and supports high TS and CS rates. However, several open questions remain regarding the role of LAMSs in this setting, including appropriate indications (e.g., type of stenosis, first-line versus refractory cases), long-term outcomes, and comparative data with other well-established techniques such as ED [43]. Finally, the economic aspect must also be considered in the decision-making process. For example, a single-center retrospective analysis of patients who underwent endoscopic treatment for benign foregut strictures evaluated the cost-effectiveness of LAMSs compared to ED, particularly in cases requiring multiple repeated procedures [74]. The study found that the cost breakeven point for the overall cohort was 3.5 dilations, while in the postsurgical subgroup, it was 2.2 dilations. These findings suggest that stent placement may offer a cost advantage over recurrent dilation beyond the third dilation [74]. In Figure 5, we have included the main advantages and limitations of each intervention to aid in clinical decision-making (Figure 5).

Future studies should prioritize the evaluation of new therapeutic strategies and a thorough assessment of current techniques in clearly defined and homogeneous patient groups. The limitations of the studies mentioned are primarily due to the considerable heterogeneity in the patient populations, especially regarding the type of surgery, anastomosis location (cervical versus intrathoracic), stenosis length, and follow-up duration. This would support the development of tailored endoscopic treatments for specific patient categories. Table 2 provides an overview of several ongoing studies (Table 2).

## 5. Conclusions

The optimal therapeutic algorithm for managing postsurgical strictures remains uncertain, with ED continuing as the first-line treatment. However, the requirement for multiple procedures increases patients’ exposure to procedural risks and contributes to substantial healthcare costs. This underscores the need for further research in this patient population to identify factors, such as surgical variables, that may serve as risk indicators to guide the selection of appropriate endoscopic management strategies. The lack of studies involving homogeneous populations, prospective designs, and direct comparisons of different endoscopic techniques hampers the development of standardized treatment protocols and the establishment of consistent endoscopic practices.

## Figures and Tables

**Table 1:** Luminal-apposing metal stent models available on the market and their technical specifications.

Stent	Manufacturer	Stent Measurements, mm		
		Internal Diameter	Flange Diameter	Stent Length
Axios	Boston Scientific, Marlborough, Massachusetts, USA	6	14	8
		8	17	8
		10	21	10
		15	24	10
		15	24	15
		20	29	10
Spaxus	Taewoong Medical, Gyeonggi-do, Republic of Korea	8	23	20
		10	25	
		16	31	
Nagi	Taewoong Medical, Gyeonggi-do, Republic of Korea	10 to 16	20	10
			20	20
			20	30
Z-EUS	M.I.Tech, Seoul, Republic of Korea	10	22 to 28	10, 30
		12		
		14		
		16		

**Table 2:** Some ongoing studies concerning the endoscopic treatment of anastomotic strictures after GI surgery.

Study Title	Design of the Study	Aim of the Study	Patients	NCT Number
Oral prednisolone in the treatment of esophageal stricture after esophageal surgery	Not specified	To evaluate: Number of participants with treatment-related AEs Incidence of treatment discontinuations, modifications, and interruptions due to AEs	Patients who develop severe esophageal strictures from 28 days after esophageal surgery	NCT02703376
Cryoablation for benign GI anastomotic strictures	Randomized controlled trial	To evaluate the impact of cryotherapy on clinical outcomes and complications for benign anastomotic strictures following esophagectomy, gastrectomy, and bariatric surgery	History of GI surgery and/or BD	NCT04372784
NKI therapy compared to usual care of recurrent esophagogastric anastomotic strictures (SAMURAI)	Multicenter randomized controlled trial	To evaluate the efficacy and cost-effectiveness of NKI followed by EBD compared to standard EBD in patients with recurrent (at least 1 to a maximum of 5 EBD sessions) esophagogastric anastomotic strictures	Patients with recurrent intra- or extrathoracic benign esophagogastric anastomotic stricture after esophagectomy	NCT04406428
Randomized controlled trial of ED: Triamcinolone Injection	Randomized controlled trial	Evaluate whether the addition of triamcinolone injection during the initial ED session decreases the need for repeat dilation procedures	Patients who present for EGD with dilation for dysphagia symptoms thought secondary to either radiation-induced stricture or anastomotic stricture	NCT01873573

AE: Adverse event; GI, gastrointestinal; BD, balloon dilation; NKI, needle-knife incision; EBD, endoscopic balloon dilation; ED, endoscopic dilation; EGD, Esophagogastroduodenoscopy.

## Data Availability

No new data was created or analyzed in this study. Data sharing is not applicable to this article.
